# Cancer-Cells on Chip for Label-Free Detection of Secreted Molecules

**DOI:** 10.3390/bios6010002

**Published:** 2016-01-15

**Authors:** Ophélie I. Berthuy, Loïc J. Blum, Christophe A. Marquette

**Affiliations:** Laboratoire de Génie Enzymatique, Membranes Biomimétiques et Assemblages Supramoléculaires—Institut de Chimie et Biochimie Moléculaires et Supramoléculaires—Université Claude Bernard Lyon 1, Villeurbanne 69100, France; berthuy@scienion.de (O.I.B.); loic.blum@univ-lyon1.fr (L.J.B.)

**Keywords:** biosensor, cell microarray, cell encapsulation, SPRi, real-time

## Abstract

In the present report, we are making the proof of concept of cell small populations (from 1 to 100 cells) spotting, culture and secretion detection on a gold surface. In order to keep the cells in a hydrated environment during the robotized micropipetting and to address different cell lines on a single chip, a biocompatible alginate polymer was used. This approach enables the encapsulation of the cell in a very small volume (30 nL), directly on the substrate and permits a precise control of the number of cells in each alginate bead. After 24 h of culture, the adherent cells are ready for surface plasmon resonance imaging (SPRi) experimentation. To enable the detection of secreted proteins, various antibodies are immobilized in an organized manner on a SPRi sensor and permitted the multiplex detection of different proteins secreted by the different cultured cell lines. Evidence of the real-time detection will be presented for Prostate Specific Antigen (PSA) and β-2-microglobulin (B2M) secreted by prostate cancer cells following induction by dihydrotestosterone (DHT). Different kinetics for the two secreted proteins were then demonstrated and precisely determined using the chip.

## 1. Introduction

Immobilizing living cells within defined arrays on platforms such as biochips holds much promise in miniaturizing assays for screening chemical libraries. Monitoring the response of several cells in a single device could provide a valuable tool in environmental monitoring, drug screening and clinical diagnosis.

During recent years, advances in cell immobilization strategies have provided the possibility to position different cells within microarrays by using various deposition and printing techniques [1], such as micro-contact printing (micro-stamping) [2] and non-contact printing like ink-jet [3]. However, immobilization of living cells is still a crucial step as their viability and activity has to be maintained by ensuring a firm fixation of all immobilized cells at the same time. Cells encapsulation is here an interesting option since cells can be maintained in a biocompatible and hydrated environment.

Hydrogels may be defined as biocompatible polymers with a high water content, which can provide a suitable environment for immobilized cells. One of the most used hydrogels for cell encapsulation is sodium alginate; it is nontoxic, and its polymerization conditions are soft enough to allow the encapsulation of diverse living cell types [4].

Once the cells are immobilized, a cell biochip might be able to produce analytical results coming from the cell activity. The requirements here are mostly sensitivity and specificity, but also real time and label free.

Surface plasmon resonance (SPR) based biosensing has been a topic drawing substantial research interests in the past decade. Promising biomedical applications of SPR have also been widely studied in the field of the detection of binding activity between cells, proteins, DNA and even small inorganic molecules [5,6,7,8]. The principle of SPR biosensors is the measurements of refractive index changes at a plane interface between two media with dielectric constants of opposite signs, a dielectric and a metal, such as gold. SPR can be excited when a wedge of polarized light is directed towards the glass side of the sensor surface under the condition of total internal reflection. The resonant angle at which a minimal intensity of reflected light occurs is a function of the local refractive index at or near the gold surface. Such refractive index changes are intimately associated with the adsorption or desorption of molecules from the surface, and thus one can expect its great potential in bio-recognition measurements [9]. This is an almost unstudied technique for biomarker detection directly in culture medium in the presence of living cells. To date, all previously established SPR based sensing platforms have been limited to detection of analyte in a prepared sample [10]. In these strategies, sampling of analytes from cell culture media, purification and pre-treatment of analytes are usually required for the purpose of cellular exocytosis and cellular signaling pathway studies [11,12]. These redundant steps are time consuming and also introduce unpredictable errors to the experiments. Therefore, it is desirable to find an alternative method for direct measurement of secretions from living cells. In that purpose, Liu *et al.* demonstrate a new concept of a SPR biosensor for biomarker study [13,14]. On the basis of integration of a mini cell culture system within the traditional SPR sensing platform, this biosensor was capable of direct measurement of VEGF biomarker secretion from living SKOV-3 carcinoma cells. However, this biosensor did not allow multiplex analysis.

**Figure 1:**
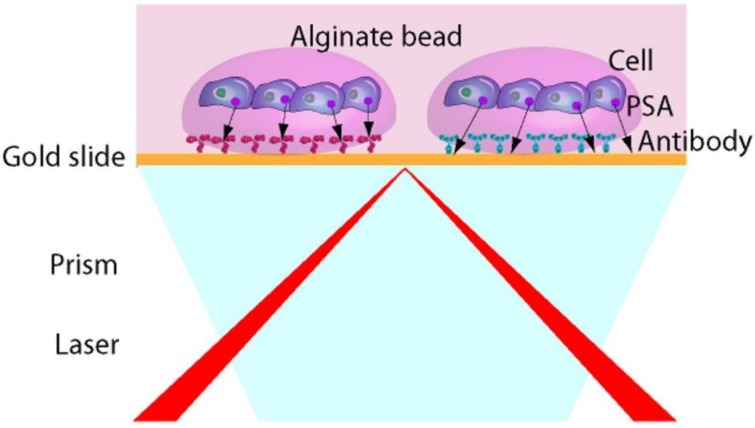
Configuration of the surface plasmon resonance imaging (SPRi) based biochip for direct measurement of secreted molecules from living cells.

In order to analyze several cell populations and detect different secreted molecules on the same chip, we have developed a novel fully automated technique for the immobilization of antibodies and cells on a SPRi biochip, using the ability of alginate hydrogel to encapsulate cells [15]. In order to demonstrate the ability of the system to detect in a real time and label free manner molecules secreted by cells, we have been working with LNCaP cells, a human prostatic carcinoma cell line. It is known that androgen receptor activity is implicated in all phases of prostate cancer and that the Prostate Specific Antigen (PSA) expression is dependent on androgen signaling pathway. In the present report, the proof of concept of the developed system (Figure 1) will be presented.

## 2. Experimental Section

### 2.1. Reagents

Anti-Prostate Specific Antigen (PSA) and Prostate Specific Antigen (PSA) were purchased from Abcam (UK). Anti-β-2-microglobulin (B2M) was obtained from Raybiotech Inc (Norcross, USA). Dulbecco’s Modified Eagle’s Medium (DMEM), Phosphate Buffered Saline (PBS), fetal calf serum (FCS), Fungizone, Penicillin/Streptomycin were purchased from Invitrogen/GibcoBRL (Cergy Pontoise, France). Streptavidin Horseradish peroxidase (HRP) labeled, luminol, hydrogen peroxide (H_2_O_2_), p-iodophenol, Calcium chloride (CaCl_2_) and 5α-Androstan-17β-ol-3-one (dihydrotestosterone, DHT) were purchased from Sigma-Aldrich (Saint Quentin Fallavier, France). Low Cross buffer was supplied by Candor Bioscience (Wangen, Germany).

### 2.2. Antibodies Spotting

**Figure 2:**
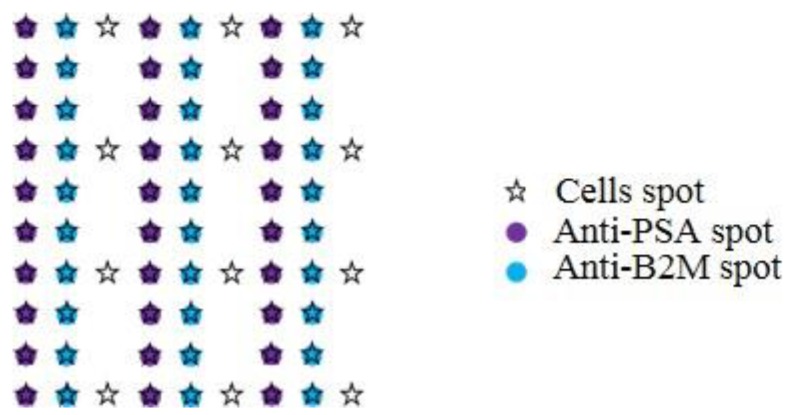
Spotting map of the biochip. Position of each antibody spots on the substrate and localized deposition of cells on top of the antibodies and on gold as negative control.

All antibodies were diluted at a final concentration of 200 µg/mL in PBS. In order to deposit a small volume (2.4 nL) of each in an organized manner onto a SPRi chip slide (Genoptics, Horiba, France), a piezoelectric spotter (sciFLEXARRAYER S1, Scienion, Germany) was used. A matrix of 60 antibody spots with a pitch of 1 mm was thereby created (Figure 2).

### 2.3. Cell Preparation

LNCaP cell line (ATCC^®^—CRL-1740™, Manassas, VA, USA) was grown on Petri dish in DMEM supplemented with 10% FCS, 1 mg/mL Fungizone and 50 U/mL Penicillin/Streptomycin at 37 °C, in humidified atmosphere containing 5% of CO_2_. After two days of cell culture, LNCaP cells were passaged by tripsinization and seeded so as to obtain the desired concentration.

### 2.4. Process for in Situ Cell Encapsulation

LNCaP cell line was re-suspended at various concentration in a 1% (*w/v*) alginate solution and 14 nL of this cell suspension solution spotted onto the antibodies spots using the piezoelectric spotter. For the *in situ* encapsulation process, 14 nL of 100 mM CaCl_2_ were spotted onto the alginate/cells spots. The encapsulation process thereby proceeded in 5 min. The substrate was then immersed in a Petri dish filled with DMEM supplemented with 2 mM CaCl_2_ at 37 °C, in a humidified atmosphere containing 5% of CO_2_. After 24 h, the substrate is assembled with a SPRi biochip.

### 2.5. ELISA for PSA Detection on Cell Culture Supernatant

In order to quantify PSA secretion by cells in classical culture conditions following induction by 100 nM of DHT, supernatant samples were collected at days D0, D+1, D+2, D+3 and D+4. A sandwich ELISA assay was performed using the following protocol: (1) coating of a microtiter plate bottom (Maxisorb, NUNC, France) with anti-PSA antibody at a concentration of 15 µg/mL overnight at 4 °C; (2) blocking with Low Cross buffer diluted at 1/5 in PBS for 1 h at 37 °C; (3) incubation of samples or standard for 1 h at 37 °C; (4) incubation of anti-PSA biotinylated at a concentration of 0.6 µg/mL for 1 h at 37 °C; (5) incubation of HRP labelled streptavidin at a concentration of 1 µg/mL at 37 °C for 1 h; (6) detection of the chemiluminescent signal in the presence of a solution composed of 220 µM luminol, 500 µM H_2_O_2_ and 200 µM p-iodophenol. The microplate was read with the luminoscope Luminoskan™ (Labsystems, Helsinky, Finland) and results analyzed thanks to the associate software (Ascent, Labsystems).

### 2.6. SPRi Experiments

SPRi experiments were performed with a SPRi lab+ (Genoptics, Horiba, France) in a batch format. The batch measurement cell was composed of a Teflon chamber with a inner volume of 400 µL. SPRi slide and SPRi prism were purchased from Horiba (Genoptics, Horiba, France). All injections to the SPRi microfluidic system were performed from the top of the open batch measurement cell. Experiments were performed directly in DMEM culture medium at room temperature.

## 3. Results and Discussion

**Figure 3:**
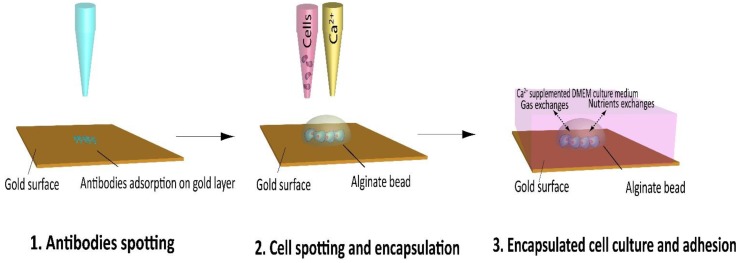
Overview of the SPRi cell chip microfabrication process using piezoelectric spotter. 1—Specific antibodies are spotted onto the SPRi chip gold surface; 2—Cell are encapsulated in alginate beads on top of the antibody spots; 3—Cells are culture in calcium supplemented medium.

The aim of the present report is to provide the proof-of-concept of the design of a new cell chip allowing real-time and label-free detection of secreted molecules to study the influence of different inducer on the androgen pathway. The fully automated fabrication process of the chip is detailed in Figure 1. The first step is to spot different antibodies on the gold surface of a SPRi chip. After one hour, cells suspension in an alginate solution are spotted, co-localized with the antibodies spot on the SPRi chip. Then, the cross-linking agent, calcium chloride is spotted onto the cells/alginate in order to encapsulate the cells in an alginate bead. This *in situ* encapsulation process permits to keep the cells in a properly hydrated environment and to physically retain them while they adhere to the chip surface. The substrate is then immersed in a Petri dish filled with culture medium supplemented with 2 mM CaCl_2_ (Figure 3).

### 3.1. Localized Antibodies Immobilization and Cell Deposition

The localized deposition of the cells and antibodies is made possible using a piezo-electric spotter. The volume deposition used with this system is very small, starting at 100 pL and allows precise localized deposition with a very short execution time. With this method, we have patterned an array of 90 cell spots onto a matrix of 30 × 2 different antibodies spots (Figure 2). Direct cell deposition in such low volume is hardly reproducible and most of the time leads to low survival rate, mainly due to the evaporation of the spotting solution (even in humidity-controlled atmosphere) [14]. To solve this problem, we have developed an automated cell deposition using a cell encapsulation technique in alginate hydrogel during cell deposition [15]. Alginate has the unique property to jellify instantaneously upon soft condition—*i.e.*, in the presence of Ca^2+^. The spotting solution is then liquid in the spotter tip, allowing for an efficient process, and the ejected drops rapidly produce a 3D gel matrix at the contact of the Ca^2+^. The process has the great advantage to be fully biocompatible and the resulting alginate hydrogel allows oxygen and nutrients exchanges between cells and culture medium while maintaining cells at a controlled position of the chip.

### 3.2. Control of Cell Numbers

**Figure 4:**
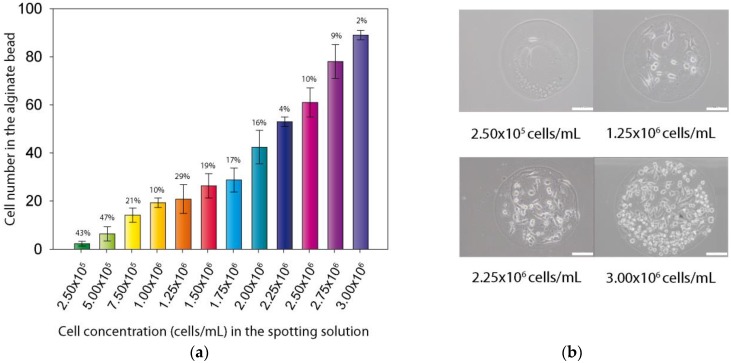
Control of cell population in alginate bead. (**a**) Number of cell per bead for each concentration of cells in spotting solution (error bars are CVs); (**b**) Optical microscopy images of four different alginate beads after 4 h of culture in beads (The white scale bar represents 100 µm).

The approach we used to design the chip enables the encapsulation of the cells in a very small volume, about 30 nL, and permits the precise control of the cell numbers in each alginate bead. Indeed, the cell number in the final alginate bead is directly related to the cell concentration in the alginate spotting solution. As can be seen in Figure 4a, a precise control of the bead cell content, from 1 to 100 can be achieved with good reproducibility, particularly for the higher cell concentrations (CVs between 2% and 10%). Nevertheless, for the lower cell concentrations, higher CVs were observed (between 10% and 47%), mostly because at these very low number of cells per spot, few cells variation in the bead had a strong impact on CV. Figure 4b also depicted the distribution of the cells in the obtained beads. As a matter of fact, even high density cell beads have the ability to confine cells within the alginate matrix.

### 3.3. SPRi Measurements

**Figure 5:**
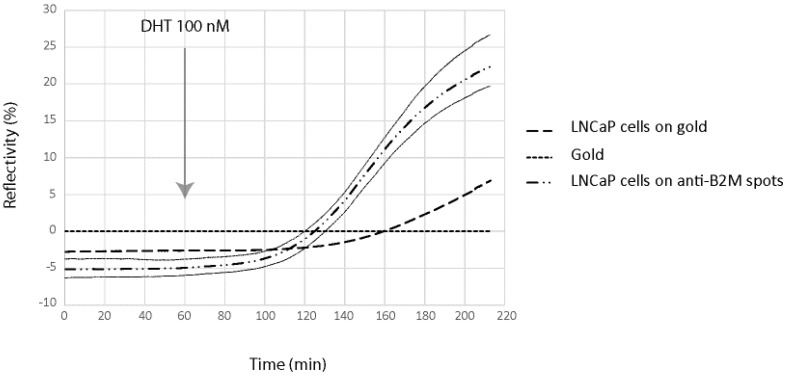
β-2-microglobulin (B2M) secretion detection using the SPRi biochip. For signal measured on anti-B2M spots, signal variation is represented by ± 2SD curves (grey lines).

**Figure 6:**
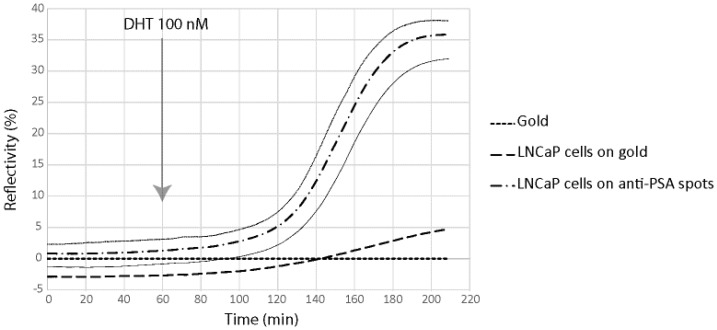
Prostate Specific Antigen (PSA) secretion detection using the SPRi biochip. For signal measured on anti-PSA spots, signal variation is represented by ± 2SD curves (grey lines).

In order to demonstrate the possibility to detect in real time and label free the kinetic of the molecules secreted by cells on the chip, we have been working on a model cell line, the human prostatic carcinoma cell line LNCaP. This cell line has the particularity to secreted PSA and B2M in response to specific stimuli, the dihydrotestosterone. LNCaP cells have been spotted at 2 × 10^6^ cells/mL in order to obtain about 50 cells per spot for SPRi experiments. In order to demonstrate direct measurement of B2M and PSA from living prostatic carcinoma cells, LNCaP cells were cultured for 24 h in alginate bead onto a SPRi slide. The slide was assembled with a SPRi prism and load into the SPRi device. The cell chamber was filled with DMEM. After one hour of stabilization, DHT 100 nM was injected and the secretion detected using both anti-PSA and anti-B2M spots (Figure 5 and Figure 6).

Four different parameters were followed using the SPR signal (Reflectivity): the SPR drift on bare gold, the SPR variation below adherent cells on gold, the SPR variation below adherent cell on anti-PSA and the SPR variation below adherent cell on anti-B2M. The results are summarized in Figure 5 and Figure 6.

**Figure 7:**
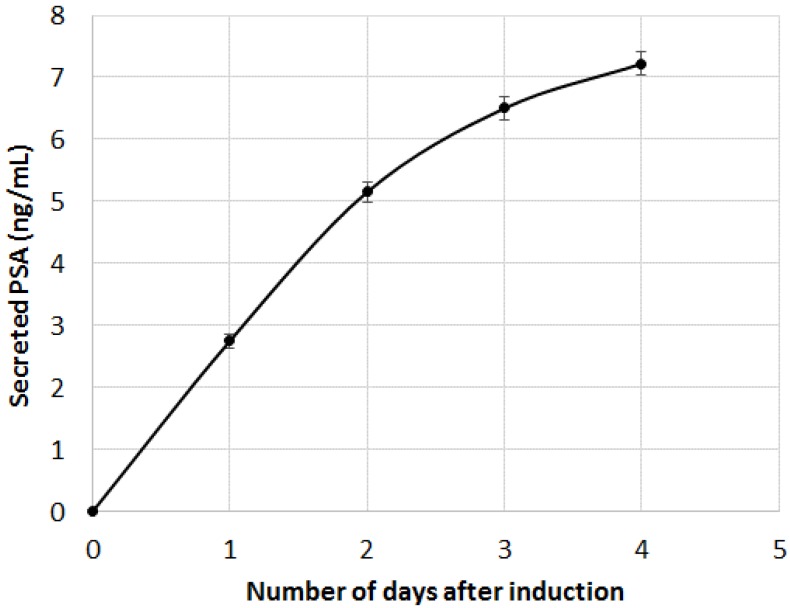
PSA immuno-detection in cell culture supernatant. DHT induction was performed at t = 0 (error bars are CVs).

As can be seen, both B2M and PSA were detected in the first 20 min following the DHT induction. The limit of detection of the two proteins were 29 pg and 58 pg for PSA and B2M, respectively (for comparison purpose, LOD using the standard ELISA was 50 pg for PSA). When compared to the secretion kinetic reachable using standard ELISA procedure on cell culture supernatant (Figure 7), the advantage of the present method looks tremendous. Indeed, using the classic immunoassay system, PSA was hardly detectable before the first day following DHT induction. Such an early detection of the secreted PSA and B2M in our microsystem was attributed to the overconcentration effect of the secreted proteins, possible only thanks to the presence of the cells in alginate beads, on top of the detecting antibodies. Indeed, a very low volume was trapped between the cell and the surface modified with antibodies, leading to a high overconcentration of the proteins.

Nevertheless, the observed secretion kinetic was a combination of the effect of the overconcentration and the saturation of the sensing layer composed of immobilized antibodies. Indeed, the measured SPR signal level off after 120–200 min of induction, more as a consequence of the antibodies binding capacity saturation than of a lowering of the secretion activity. As a consequence, the long term kinetic observed using the classical ELISA system cannot be reached using the present microsystem.

### 3.4. Quantitative Analysis

In order to evaluate the quantity of protein secreted by each encapsulated spot, reflectivity variations were converted to amount of molecules per unit area (in pg/mm^2^) using the following equation (equation given by Genoptix):

$$\tau =\frac{\Delta {\mathrm{RL}}_{\mathrm{ZC}}}{S_{\text{P,R}}\;\delta n/\delta c}$$

where ΔR is the reflectivity variation in percentage, L_ZC_ = 1.02 × 10^−4^ mm (depth of penetration of the plasmon wave), S_P,R_ = 2.25 × 10^3^ %/RIU (sensitivity of the SPR as a percentage per unit of refractive index) and Δn/δc = 1.9 × 10^−10^ mm^3^/pg.

The highest reflectivity variations are 35% and 15% for PSA and B2M, respectively, corresponding to 8351 pg/mm^2^ of PSA and 3579 pg/mm^2^ of B2M. These are high surface concentration of protein and further calculations are needed to evaluate the compatibility of these numbers with the size of the antibody spots.

The antibody spots have an average diameter of 250 microns which corresponds to a surface of 49,062.5 μm². It is then possible to estimate the amount of protein per spots: 409 pg for PSA and 176 pg for B2M. Using the Avogadro constant (N_A_ = 6.02214129 × 10^23^ mol^−1^) and the molecular weight of the two proteins, it is then possible to obtain the actual number of molecules per spot. The PSA has an average molecular mass of 28 kDa, leading to 15 × 10^−15^ mole of PSA, corresponding to 9 × 10^9^ molecules. B2M has an average molecular weight of 12 kDa, corresponding to 15 × 10^−15^ mole or 9 × 10^9^ molecules per spot.

The two proteins have a diameter of about 20 Å (2 nm) giving a projected area of 3.14 nm^2^ (3.14 × 10^−6^ μm^2^). Using the mean spot area of 49,062.5 μm^2^ it is then possible to calculate a maximum number of protein per spot of 1.6 × 10^10^ molecules. The amount of protein per spot calculated previously using the saturation reflectivity are then fully compatible with an almost full coverage of the antibody spots.

## 4. Conclusions

A cell-on-chip analytical system for the label free detection of secreted molecules in real time has been developed and the proof of concept of the detection of two secreted proteins validated. The architecture of the system, with its alginate beads trapping living cells on top of antibody spots leads to the overconcentration of the secreted proteins, enabling the early detection of secreted molecules within minutes of induction. Different early kinetics for the two secreted proteins, B2M and PSA, were then demonstrated and precisely determined using this novel technique.

From a biological point of view, SPR results demonstrated that PSA, the most used biomarker of prostate cancer, and B2M were secreted in similar amounts. B2M secreted protein level might then be high enough to be considered as a new biomarker of prostate cancer [16,17]. There is no doubt that our system will, in the near future, be applied to more multiplexed and complex biological secretion systems for which kinetic data are at the moment not reachable using standard cellular biology methods.
